# Metabolism Profile of Mequindox in Sea Cucumbers In Vivo Using LC-HRMS

**DOI:** 10.3390/antibiotics11111599

**Published:** 2022-11-11

**Authors:** Xin Mao, Xiaozhen Zhou, Jun He, Gongzhen Liu, Huihui Liu, Han Zhao, Pengjie Luo, Yongning Wu, Yanshen Li

**Affiliations:** 1 Department of Marine Product Quality and Safety Inspection Key Laboratory, Yantai University, Yantai 264005, China; 2 College of Agriculture and Forestry, Linyi University, Linyi 276000, China; 3 Shandong Marine Resource and Environment Research Institute, Yantai 264006, China; 4 NHC Key Laboratory of Food Safety Risk Assessment, Chinese Academy of Medical Science Research Unit (2019RU014), China National Center for Food Safety Risk Assessment, Beijing 100017, China

**Keywords:** mequindox, metabolites, metabolic pathway, sea cucumbers, deoxidation, carbonyl reduction

## Abstract

In this work, the metabolism behavior of mequindox (MEQ) in sea cucumber in vivo was investigated using LC-HRMS. In total, nine metabolites were detected and identified as well as the precursor in sea cucumber tissues. The metabolic pathways of MEQ in sea cucumber mainly include hydrogenation reduction, deoxidation, carboxylation, deacetylation, and combinations thereof. The most predominant metabolites of MEQ in sea cucumber are 2-iso-BDMEQ and 2-iso-1-DMEQ, with deoxidation and carbonyl reduction as major metabolic pathways. In particular, this work first reported 3-methyl-2-quinoxalinecarboxylic acid (MQCA) as a metabolite of MEQ, and carboxylation is a major metabolic pathway of MEQ in sea cucumber. This work revealed that the metabolism of MEQ in marine animals is different from that in land animals. The metabolism results in this work could facilitate the accurate risk assessment of MEQ in sea cucumber and related marine foods.

## 1. Introduction

Quinoxalines, a class of synthetic quinoxaline veterinary drugs with a Quinoxaline 1,4-di-N-oxides structure (QdNOs), were reported with broad-spectrum antimicrobial activity [1]. QdNOs have been applied as food additives in farm animal husbandry. As a major QdNO, Mequindox (3-methyl-2-quinoaxlinacetyl-1,4-dioxide, MEQ) was developed in the 1980s. Due to its high antibacterial activity, MEQ was used as an additive in swine and chicken feed [2]. Recently, QdNOs were reported with potential carcinogenicity and mutagenicity [3,4]. In particular, carbadox (CBX) and olaquindox (OLA), two members of QdNOs, were banned by the European Commission (EC) [5]. Deriving from the same family of QdNOs with a corresponding basic structure, MEQ has attracted increasing attention due to corresponding toxic risk, including induced apoptosis, carcinogenicity, DNA damage, etc. [6,7].

It was reported that MEQ could be easily metabolized after ingestion by animals, and some of the metabolites were detected and their structure identified in previous metabolic investigations in chicken and swine [8,9]. N → O group reduction and carboxylation were the major metabolic pathways in farm animals [10]. In farm animals, the major N → O group reduction metabolites were 1–desoxymequindox (1–DMEQ) and 1,4–bisdesoxymequindox (BDMEQ). Moreover, carboxylation metabolites and hydroxylation metabolites were also detected and identified. However, these metabolic investigations and the metabolites identified were mainly in land farm animal tissues and related products. There are few reports regarding metabolism investigations of QdNOs in aquatic animals. The analytical methods for QdNOs and metabolites detection in aquatic animals and related products were mainly based on metabolic results in land farm animals and the metabolites identified in chicken and swine [11,12]. The potential metabolic enzyme between land farm animals and aquatic animals might lead to different metabolic mechanisms [13,14]. To the best of our knowledge, there have been no related comprehensive metabolic investigations of MEQ in sea cucumber.

As a high-nutrition and high-value marine food, sea cucumbers have been farmed and exploited for commercial use as food and functional food [15,16]. Recently, sea cucumbers have been investigated and processed for different functional foods, and the sea cucumber industry is becoming one of the most important food commodities around the globe for health purposes [17,18]. Bioactive compounds in sea cucumbers were detected and reported with antioxidant, antihypertensive, anti-inflammatory, anticancer, antimicrobial activities [19]. Mequindox has been widely applied and used as a feed additive and antibacterial medicine in the sea cucumber farming industry [20]. Considering the extensive application of sea cucumber in food and health-care food industry, the identification of potentially hazardous compounds in sea cucumber appears to be necessary.

In this metabolism investigation, the major metabolites and metabolic pathways of MEQ in sea cucumber were examined. This work could provide the basis and target compound for further control of sea-cucumber-related food safety.

## 2. Results and Discussion

### 2.1. Optimization of Sample Preparation

**Figure 1:**
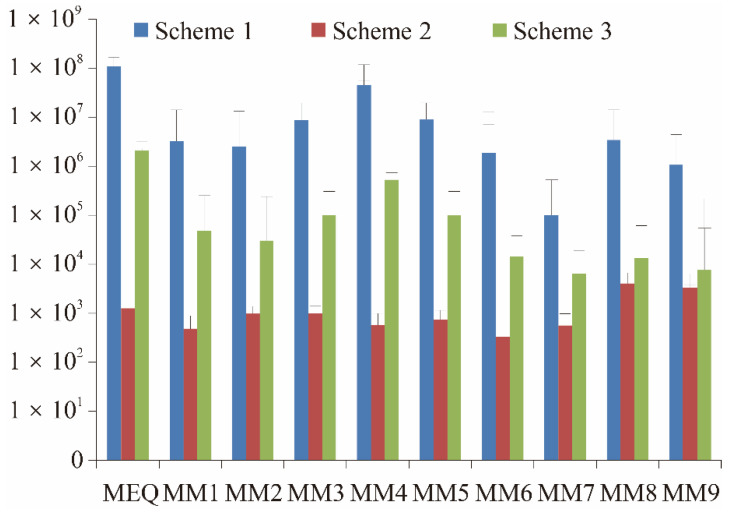
Optimization of extraction efficiency with three different schemes (*n* = 6).

In order to obtain more comprehensive metabolites, the first and most important step is target compounds and metabolites extraction. In this work, three schemes were optimized according to the previous literature [21,22,23], with some modification. An experiment group sample and bland control of 2.00 ± 0.02 g were weighed into a 50 mL polypropylene centrifuge tube, respectively. In total, 10 compounds, including MEQ and 9 metabolites, were evaluated in different pretreatment schemes. By comparing the extraction effects of three different schemes, it was obvious that Scheme 1 led to the most optimized results with the highest abundance of all target compounds (Figure 1). Additionally, Scheme 1 was used for MEQ and metabolites pretreatment of sea cucumber.

### 2.2. Identification of Metabolites in Sea Cucumber

**Figure 2:**
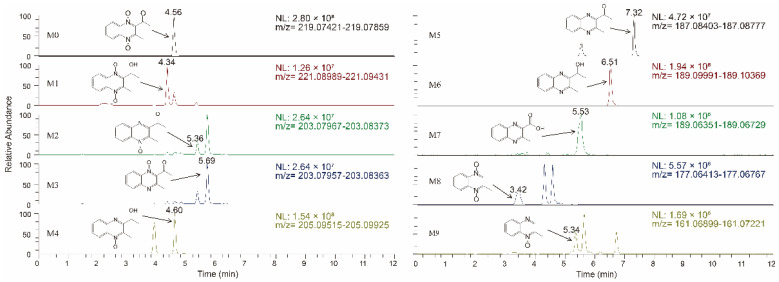
Accurate extracted ion chromatograms (EICs) of mequindox and its major metabolites in sea cucumber in vivo.

**Figure 3:**
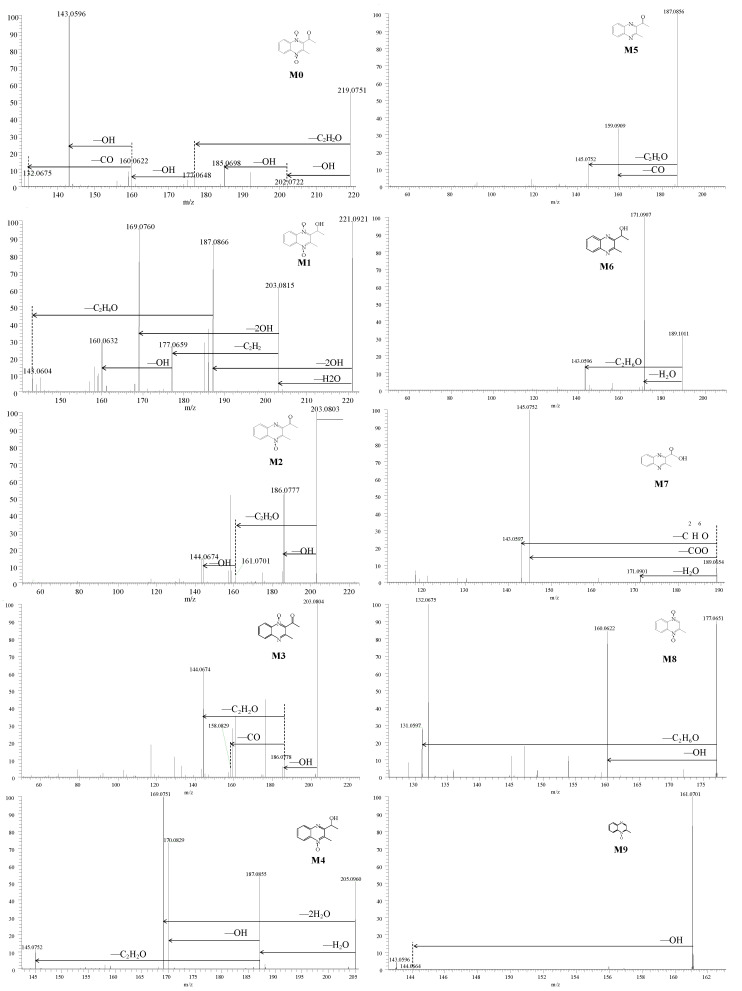
Accurate MS2 spectra and major proposed neutral loss of MEQ and metabolites detected in DDA mode. The MS2 spectra were produced by collision-induced dissociation (CID) of the selected precursor ions with different energies (20, 40, 70).

After administration, all experimental and control group samples were simply pretreated according to Scheme 2 and determined by HRMS. According to the elemental composition of MEQ and the accuracy mass difference value between the metabolites and MEQ, the elemental composition of metabolites could be speculated. In total, nine metabolites were detected and identified in sea cucumber in vivo. The predicted elemental compositions ([M + H]^+^), mass errors, retention times (RT), fragmentation ions, and relative percentages of peak area are presented in Table 1. The errors between the measured and predicted masses were within less than 6 ppm, which demonstrated the high mass accuracy of the instrument. The chromatograms of MEQ and metabolites are shown in Figure 2, and the fragmentation ions and potential neutral losses are shown in Figure 3.

#### 2.2.1. Performance of MEQ in Mass Spectrum

MEQ is eluted at a retention time of 4.56 min with protonated molecule ([M + H]^+^) at *m/z* 219.0764. An MS/MS spectrum of MEQ is acquired under collision-induced dissociation (CID) with fragmentation ions of *m/z* 143.0596, 185.0698, 160.0622, 177.0648, 202.0722, and 132.0675. The predicted elemental compositions, observed and calculated masses, and mass errors of protonated MEQ and its fragmentation ions are presented in Figure 3.

From the figure, it can be concluded that the loss of the OH radical from MEQ led to formation of a product ion at *m/z* 202.0722 and 185.0698; further loss of the C_2_H_2_O side chain resulted in *m/z* 143.0596. In other words, protonated MEQ led to loss of the C_2_H_2_O side chain to form the fragment *m/z* 177.0648; further loss of the OH radical led to formation of *m/z* 160.0622. Subsequently, the fragment ion at *m/z* 160.0622 lost the CO radical to form the product radical at *m/z* 132.0675.

#### 2.2.2. Metabolite MM1

Metabolite MM1 is eluted at a retention time of 4.34 with a protonated molecule at *m/z* 221.0921. In the accurate MS2 spectra of MM1, the fragment ions are at *m/z* 169.0760, 187.0866, 203.0815, 160.0632, 177.0659, and 143.0604. The protonated MM1 and product ion *m/z* 187.0866 are 2 Da higher than MEQ and the metabolites at *m/z* 185.0709, suggesting a hydrogenation reduction metabolite with two hydrogen additions. The fragment ion at *m/z* 187.0866 is 34 Da lower than the protonated molecule of MM3 at *m/z* 221.0920, showing that the *m/z* 187.0709 is formed by the loss of the 2 OH group, indicating that two N → O groups still exist in MM1 as MEQ. In addition, the fragment ions at *m/z* 169.0760 and 203.0815 are neutral losses of H2O from the fragment at *m/z* 187.0866 and protonated MM1 with 18 Da mass shift, which suggests that hydrogenation takes place in the acetyl group of a further side chain. The fragment at *m/z* 143.0603 is formed by losing the C_2_H_4_O of the side chain from the fragment at *m/z* 187.0866 with 44 Da mass shift. The fragment at *m/z* 177.0659 is formed by losing the side chain of fragment *m/z* 203.0815 and further loss of the OH group to form fragment *m/z* 160.0632. From the mass spectra, MM1 is considered as 2-isoethanol-mequindox (2-iso-MEQ).

#### 2.2.3. Metabolites MM2, MM3, and MM4

Metabolites MM2 and MM3 are eluted at a retention time of 5.36 min and 5.69 min with protonated molecules at *m/z* 203.0803 and 203.0804, which are 16 Da lower than MEQ. For MM2, the MS2 spectra of fragments at 203.0803, 186.0777, 161.0701, and 144.0674 are 1 Da higher than fragments of MEQ, indicating a similar structure between MM2 and MEQ. According to the MEQ mass spectra, the coordination bond oxygen could be eliminated by the OH group with a neutral loss at 17 Da. For MM3, the fragment ion *m/z* 186.0778 is 17 Da lower than the protonated molecule by losing the OH group. Fragment ions *m/z* 144.0674 and *m/z* 158.0829 are further eliminated from the C_2_H_2_O and CO groups, respectively. From the mass spectra, MM2 and MM3 are identified as the N→O group reduction metabolites of MEQ. The amount of MM3 is higher than MM2 in the chromatogram. From the structure of MEQ, the side chain at position 2 could enhance the steric hindrance of oxygen at position 1. The deoxidation reaction at position 4 is easier than at position 1. Therefore, MM3 is identified as 4-desoxy-MEQ (4-DMEQ), while MM2 is identified as 1-desoxy-MEQ (1-DMEQ).

Metabolite MM4 is eluted at a retention time of 4.60 with protonated molecule at *m/z* 205.0960. MM4 is 2 Da higher than *m/z* 203.0816, indicating a hydrogenation reduction metabolite of desoxy-MEQ. The natural loss of 17 and 18 Da might result from the coordination bond oxygen and the hydroxyl group in the structure. Considering that the coordination bond oxygen at position 1 is much more stable than at position 4 as MM3, therefore, MM4 is considered as 2-isoethanol-1-desoxy-MEQ (2-iso-1-DMEQ).

#### 2.2.4. Metabolites MM5, MM6, and MM7

Metabolite MM5 is eluted at a retention time of 7.32 with protonated molecule at *m/z* 187.0856, which is 32 Da lower than MEQ. In addition, the neutral loss of 28 Da is detected in MS2 spectra, indicating a carbonyl group in the structure. MM5 is considered as a bisdesoxy-MEQ metabolite (BDMEQ).

Metabolites MM6 and MM7 are eluted at a retention time of 6.52 min and 5.51 min with protonated molecule at *m/z* 189.1011 and *m/z* 189.0654. Based on the varied *m/z* value, the elemental composition of MM6 and MM7 might be different. From the Xcalibur platform, the fragment of *m/z* 189.1011 (MM6) is recommended as C_11_H_13_N_2_O, while *m/z* 189.0654 (MM7) is C_10_H_9_N_2_O_2_. In the MS2 spectra of MM6, the neutral losses of 18 Da and 46 Da in MM6 are detected by loss of the H_2_O and C_2_H_6_O side chain. In the MS2 spectra of MM7, the neutral loss of 44 Da is recommended as the loss of the COO group, indicating a carboxyl group in MM7. Therefore, MM6 is considered as 2-isoethanol bisdesoxy-MEQ (2-iso-BDMEQ), and MM7 is considered as 3-methyl-2-quinoxalinecarboxylic acid (MQCA).

#### 2.2.5. Metabolites MM8 and MM9

Metabolite MM8 is eluted at a retention time of 3.42 min with protonated molecule at *m/z* 177.0651. MM8 is 42 Da lower than MEQ, indicating the loss of the acetyl group in the structure. In the MS2 spectra, fragment ions *m/z* 160.0622 and 143.0606 are formed with neutral losses of 17 and 34 Da, which is similar to MEQ losing two N→O groups in turn. Therefore, MM8 is considered as deacetyl-MEQ.

Metabolite MM9 (5.35 min) with protonated molecule at *m/z* 161.0706 is 16 Da lower than MM8. Different from MM8 in the MS2 spectra, only a 17 Da neutral loss is detected without 34 Da. According to the easy deoxidation reaction at position 4, MM9 is considered as deacetyl-1-desoxy-MEQ (Deacetyl-1-DMEQ).

### 2.3. Metabolic Pathway of MEQ in Sea Cucumber In Vivo

**Figure 4:**
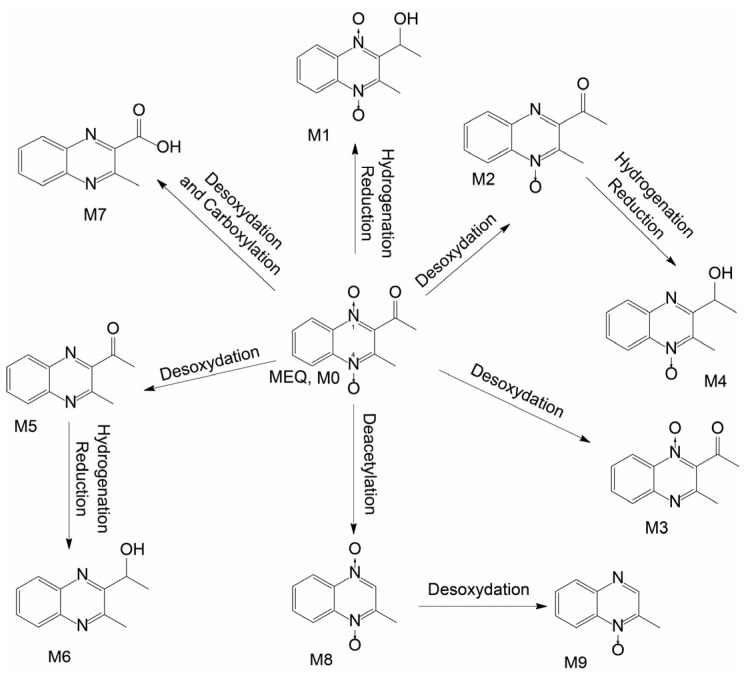
Proposed metabolic pathways of MEQ and metabolites in sea cucumber in vivo (hydrogenation reduction, deoxidation, carboxylation, deacetylation, and combinations thereof).

The results show that mequindox can be metabolized in sea cucumber after administration. In total, nine metabolites of MEQ are detected and identified by HRMS in sea cucumber. The metabolic pathways of MEQ in sea cucumber mainly include hydrogenation reduction, deoxidation, carboxylation, deacetylation, and combinations of these metabolic pathways (Figure 4). Table 1 shows the relative percentage of metabolites as well as mequindox in sea cucumber in vivo. The relative percentages of these targets are estimated on the basis of peak areas in HRMS. From the table, M6 and M4 are the predominant metabolites of MEQ in sea cucumber, accounting for 36.6% and 17.4% of all MEQ and metabolites. The percentages are: M5 (5.2%), M3 (3.4%), M1 (1.9%), M2 (1.1%), M8 (0.5%), M7 (0.4%), and M9 (0.2%).

In the previous literature on MEQ metabolism investigations, the predominant metabolite of MEQ in rat is 1-DMEQ, which takes almost half of total MEQ and metabolites, followed by BDMEQ and 3-hydroxymethyl-1-DMEQ. In chicken, the predominant metabolite is 1-DMEQ, as in rat, followed by BDMEQ, 2-isoethanol-1-DMEQ, and 2′-hydroxyacetyl-1-DMEQ. According to a MEQ metabolism study in pig, the predominant metabolite is 2-isoethanol-1-DMEQ. Additionally, 1-DMEQ, 2-isoethanol-MEQ, and BDMEQ are also reported as major metabolites. From the literature, N→O group reduction is the most predominant metabolic pathway of MEQ metabolism in land animals [21]. Different from land animals, in the metabolism investigation of MEQ in sea cucumber, the most predominant metabolic pathways are deoxidation and carbonyl reduction, with M6 (2-isoethanol-BDMEQ) and M4 (2-isoethanol-1-DMEQ) as major metabolites. In particular, carboxylation is also detected as a major metabolic pathway, with MQCA (M7) as one of the metabolites of MEQ. MQCA was reported as a residue marker of olaquindox in land farm animals in previous literature works [24,25]. This work first reported MQCA as one of the metabolites of MEQ in sea cucumber.

## 3. Materials and Methods

### 3.1. Chemicals and Reagents

MEQ (purity > 98%) was purchased from A Chemtek, Inc. (Woburn, MA, USA). Sea cucumber samples were purchased from the local market. Acetonitrile, Methanol, formic acid, and ethyl acetate (HPLC grade) were purchased from Dima Technology Inc. (Muskegon, MI, USA). Metaphosphoric acid (analytical reagent) was purchased from Shanghai Macklin Biochemical Co., Ltd. (Shanghai, China). Hydrochloric acid (analytical reagent) was purchased from Yantai Sanhe Chemical Reagent Co., Ltd. (Yantai, Shandong, China). Oasis HLB cartridges (3 mL/60 mg) were purchased from Agilent Technologies (Palo Alto, CA, USA).

### 3.2. Apparatus

Ultimate 3000 pumping system coupled with a quadrupole-Orbitrap UHPLC-Q/Exactive Plus was obtained from Thermo Fisher Scientific (Bremen, Germany). A QL-901 vortex mixer was purchased from Haimen Kylin-Bell Lab Instruments Co., Ltd. (Haimen, Jiangsu, China). An HH-6 digital display constant temperature water bath was purchased from Changzhou GuoHua Electric Appliance Co., Ltd. (Changzhou, Jiangsu, China). A KQ5200 ultrasonic cleaner was purchased from Kunshan Ultrasonic Instruments Co., Ltd. (Jiangsu, China). An H2050R centrifuge was obtained from Changsha High-tech Industrial Development Zone Xiangyi Centrifuge Instrument Co., Ltd. (Changsha, Hunan, China). Syringe filters (0.2 μm) were obtained from Waters (Milford, MA, USA). Air-pump nitrogen evaporator was obtained from Hangzhou mio Co., Ltd. (Hangzhou, China).

### 3.3. Sample Preparation

Live sea cucumbers (150 ± 10 g, 3 years old) were obtained from local aquaculture and raised for 7 days to ensure they were MEQ free. Sea cucumbers were divided into the experimental group and the control group with six replicates each. Sea cucumbers in the experimental group were raised in sea water with 4 mg/L MEQ, while the control group were raised in sea water free of MEQ for 24 h. Afterward, sea cucumbers in the experimental and the control group were collected. Each sea cucumber sample was homogenized with a blender machine for further treatment. For sea cucumber preparation, 2.00 g ± 0.02 g experiment samples and blank control samples were weighed into a 50 mL polypropylene centrifuge tube, respectively. Detailed preparation procedures were optimized with three different schemes based on previous literature works [21,22,23], and each scheme was run in six repetitions. In Scheme 1, each sample was extracted with 5 mL of methanol by vortexing for 5 min and supersonic extraction for 2 min. Each sample was centrifuged at 10,000× *g* for 15 min at 4 °C in a refrigerated centrifuge, and the supernatant was transferred into another 50 mL polypropylene centrifuge tube. The residues were re-extracted with 5 mL of ethyl acetate containing 0.1% formic acid. The supernatant was combined after centrifugation and taken to dry under a stream of nitrogen at 45 °C. Then, the residue was re-dissolved with 1 mL of methanol–water (50:50, *v/v*) and filtered through a 0.22 μm syringe filter into auto-sampler vials for HRMS analysis. In Scheme 2, 20% metaphosphoric acid was added to each sample and settled in a water bath at 55 °C for 30 min for acidolysis and extraction. After centrifugation at 10,000× *g* for 15 min at 4 °C in a refrigerated centrifuge, the supernatants were purified with an OASIS HLB column. In Scheme 3, 2 mol/L hydrochloride acid was added, and each sample was put in a water bath at 55 °C for 30 min for acidolysis and extraction. After the water bath and centrifugation at 10,000× *g* for 15 min at 4 °C in a refrigerated centrifuge, the supernatant was purified with an OASIS HLB column. Each sample was filtered through a 0.22 μm syringe filter into auto-sampler vials for HRMS analysis.

### 3.4. Instrumental Conditions

The HRMS analysis was operated in a positive mode. Samples were analyzed using liquid chromatography coupled with Q/Exactive high-resolution mass spectrum (HRMS). Samples were separated with a Hypersil GOLD C18 column (100 mm × 2.1 mm, i.e., 1.9 μm) by a gradient elution program with water containing 0.1% formic acid as mobile phase A and acetonitrile containing 0.1% formic acid as mobile phase B. The flow rate of the mobile phase was 0.3 mL/min with injection volume at 5 μL. The gradient elution program was as follows: 0–2 min 95% A, 2–3 min 95–75% A, 3–4 min 70–60% A, 4–5.5 min 60–45% A, 5.5–7.5 min 45–5% A, 7.5–9 min 5–95%, and 9–12 min 95% A.

The mass spectrum parameters for HRMS were operated in a data-dependent acquisition (DDA) mode with negative heated electrospray ionization (HESI+). The parameters for full mass scan in DDA were settled at a resolution of 70,000 FWHM, scan range of *m/z* 120–900, automatic gain control (AGC) target of 3.0 × 10^6^, and maximum ion implantation time (Maximum IT) of 64 ms. The ddMS2 parameters in the DDA mode were settled at a resolution of 17,500 FWHM, TOP N = 5, maximum IT 64 ms, isolation window 1.2 *m/z*, and normalized collisional energy (NCE) at 20, 40, and 60 eV.

### 3.5. Data Processing

In this study, Thermo’s in-house software Xcalibur was used to demonstrate the high mass accuracy of the instrument by comparing the measured and predicted masses of targets [26]. Simultaneously, the metabolites could be detected and identified by comparing the precursor, fragment ions, and neutral loss between the experimental and control samples. The mass tolerance window was set to 10.0 ppm. The smoothing setting was enabled. The smoother type Gaussian was selected with 7 smoothing points.

## 4. Conclusions

In general, the metabolism behavior of MEQ in sea cucumber in vivo was investigated in the current study. The result shows that, in total, nine metabolites were detected and identified, as well as the precursor. Different from the metabolism result in land farm animals, the most predominant metabolites of MEQ in sea cucumbers were 2-iso-BDMEQ and 2-iso-1-DMEQ, with deoxidation and carbonyl reduction as major metabolic pathways in sea cucumber. In particular, this work first reported MQCA as a metabolite of MEQ, and carboxylation is a major metabolic pathway of MEQ in sea cucumbers. This work reveals that the metabolism of MEQ in marine animals is different from that in land animals. The metabolism results in this work could facilitate the accurate risk assessment of MEQ in sea cucumber and related marine foods.

## Figures and Tables

**Table 1:** Retention times (RT), predicted elemental compositions ([M + H]^+^), mass errors, major fragmentation ions, and relative percentages of protonated MEQ and metabolites.

Compound Name	RT (min)	[M + H]^+^ (*m/z*)	Predicted Composition ([M + H]^+^)	RDB	Error ppm	Major Fragment Ions	Relative Percentage
MEQ, M0	4.56	219.0764	C_11_H_11_O_3_N_2_	7.5	0.143	143.0596, 185.0698, 160.0622, 177.0648, 202.0722, 132.0675	33.3%
2-iso-MEQ, M1	4.34	221.0921	C_11_H_13_O_3_N_2_	6.5	0.367	169.0760, 187.0866, 203.0815, 160.0632, 177.0659, 143.0604	1.9%
1-DMEQ, M2	5.36	203.0803	C_11_H_11_O_2_N_2_	7.5	0.817	203.0803, 186.0777, 161.0701, 144.0674	1.1%
4-DMEQ, M3	5.69	203.0804	C_11_H_11_O_2_N_2_	7.5	0.718	186.0778, 144.0674, 158.0829	3.4%
2-iso-1-DMEQ, M4	4.60	205.0960	C_11_H_13_O_2_N_2_	6.5	0.370	169.0751, 187.0855, 205.0960, 171.0900, 145.0752	17.4%
BDMEQ, M5	7.32	187.0856	C_11_H_11_ON_2_	7.5	−5.503	159.0909, 145.0752	5.2%
2-iso-BDMEQ, M6	6.52	189.1011	C_11_H_13_ON_2_	6.5	−5.762	171.0907, 143.0598	36.6%
MQCA, M7	5.51	189.0654	C_10_H_9_O_2_N_2_	7.5	−2.507	145.0752, 143.0597, 171.0901	0.4%
deacetyl-MEQ, M8	3.42	177.0651	C_9_H_9_O_2_N_2_	6.5	2.010	160.0622, 143.0606	0.5%
Deacetyl-1-DMEQ, M9	5.35	161.0706	C_9_H_9_ON_2_	6.5	−4.963	144.0644	0.2%

## Data Availability

Not applicable.
